# Cluster-Disjoint Multipath Routing Protocol for Real-Time and Reliable Packet Transmission in Wireless Sensor Networks

**DOI:** 10.3390/s23218876

**Published:** 2023-10-31

**Authors:** Sangdae Kim, Hakgyun Roh, Kwansoo Jung

**Affiliations:** 1 Department of Medical Information Engineering, Soonchunhyang University, Asan 31538, Republic of Korea; sdkim.mie@sch.ac.kr; 2 Department of Fintech, Daejeon University, Daejeon 34519, Republic of Korea; hg.roh@dju.ac.kr

**Keywords:** Wireless Sensor Networks (WSNs), multipath routing protocol, cluster-disjoint, real time, reliability

## Abstract

Multipath routing protocol, which is one of the typical schemes to improve end-to-end transmission success ratio in Wireless Sensor Networks (WSNs), exploits two or more paths. However, collisions and interference might occur when each path is adjacent. To prevent this problem, they construct node- or link-disjointed paths. Although the paths could prevent the above-mentioned problem, it still has an issue in the area of time delay. To exploit the disjointed paths, pre-path construction is required. In addition, a lot of time is incurred to recover the whole path when the part of the path is damaged. This phenomenon adversely affects the end-to-end transmission success ratio and transmission time. To alleviate this problem, we propose a cluster-disjoint multipath routing protocol. The proposed scheme divides the whole network into grid clusters of a certain size in the network initialization phase. Each sensor could transmit packets through the pre-constructed cluster structure without the path construction phase. Also, even if any node fails, it could be easily replaced by other nodes within the cluster region. The simulation results show that the proposed scheme has an advantage in terms of transmission delay and end-to-end transmission success ratio compared to existing multipath routing protocols.

## 1. Introduction

A Wireless Sensor Network (WSN) consists of sensor nodes with limited power supply, restricted communication range, and constrained computational capability [1]. To overcome these limitations, various routing protocols have been proposed, and in particular the multipath routing protocol is exploited to provide fault-tolerant routing, congestion control, and Quality of Service (QoS) support [2,3,4]. Namely, multipath routing could improve load balancing and reliability of packet delivery.

However, if a node participates in multiple paths, that is, parts of multiple paths overlap, the above advantages may be lost. To prevent the path overlap problem and achieve the above-mentioned purpose of a multipath, the source node that detected some event transmits a path construction message to the sink node. In the process of message relay, the nodes which received the construction message are chosen as members of the path. After the construction of one path, if the member nodes receive a path construction message to construct another path, the nodes ignore the message. The message is transmitted to a non-member node, and it is chosen as the member node of another path. Through these processes, a node is prevented from participating in different paths. The routing protocol exploiting a multipath constructed by these processes is called a node-disjoint multipath routing protocol [5,6,7,8].

The node-disjoint multipath routing protocol is suitable for load balancing to deal with the congestion control. However, since each path is adjacent, it is difficult to transmit packet exploiting multipaths simultaneously to improve end-to-end transmission success ratio due to collisions and interference between adjacent nodes. Therefore, multipaths are constructed at a certain distance between each path to avoid collisions and interference that may occur between nodes. This certain distance is usually determined by the transmission range of a node, and it is called a guardband. Since each path transfers a packet with the guardband in between, unlike nodes in the node-disjoint multipath routing protocol, it is not affected by collision and interference [9,10,11]. However, traditional multipath routing protocols should construct the paths before packet transmission to the sink node, and even if a part of the path is broken, a new path should be constructed. This phenomenon adversely affects end-to-end transmission success ratio and transmission time, which are important factors in WSNs.

Therefore, in this paper, we propose a cluster-disjoint real-time and reliable packet transmission scheme. In the proposed scheme, the network is divided into a grid cluster of a certain size according to the transmission range at the network initialization phase. In the phase, each cluster is numbered by an (x,y)-coordinate. After the initialization phase, the protocol operates as follows:

- When any source node detects an event, the source node delegates the packet to the cluster head to which it belongs. The cluster head transmits the packet to the next cluster closest to the sink node according to the cluster information calculated in the network initialization phase.
- The node inside the next cluster that received the packet delegates the packet to its cluster head, and the cluster head transmits the packet to its next cluster. In this process, each cluster head caches information on the cluster selected as the next cluster. Through this process, one shortest path is constructed.
- If more than one path is required, the cluster head repeats the above process by selecting the next cluster closest to the sink node, and additional clusters according to the number of paths are required.

That is, since the cluster to which the packet is transmitted is determined simultaneously with packet transmission, no additional time to construct a path is required. In addition, in the case of path failure, since other nodes in the cluster could be delegated the role of existing nodes without any process, it takes significantly less time than in the case of existing schemes in terms of path recovery.

The remainder of this paper is organized as follows. In Section 2, we describe the existing node-/radio-disjoint multipath routing protocols. In Section 3, we explain the proposed cluster-disjoint multipath routing protocol for real-time and reliable packet transmission. The performance evaluation results are provided in Section 4. Finally, the proposed scheme and simulation results are summarized in Section 5.

## 2. Related Work

In this section, we briefly summarize the existing node-disjoint [7,8]/radio-disjoint [10,11] multipath routing protocols.

NDRECT [7] is a node-disjoint multipath scheme to support real-time traffic, which consists of two stages of the discovery phase and the path establishment phase. In the discovery phase, the source node floods the route request (RREQ) message throughout the network to discover the sink node. In this process, intermediate nodes categorize neighboring nodes through RREQ messages received from different neighbors as predecessors, same level, and successors, and are tagged with different values depending on the number of RREQ messages received from the different predecessors. The RREQ message contains information for route selection, and to mitigate flooding overhead, the intermediate node aggregates the RREQ message received from the neighboring node and forwards only one RREQ message. Through the above process, the sink node which received the RREQ message can select the most appropriate peers and return a route reply (RREP) message to the source node. The RREP message includes necessary information to select the next node to reach the source node. This RREP message, which arrives at the source node, contains information on the nodes that it has been through. Finally, the source node could transmit a packet through a node-disjoint multipath to the sink by exploiting this information.

FD-AOMDV [8] is a multipath routing protocol which exploits a node-disjoint approach to find alternate paths between the source and sink nodes. To construct a multipath, the source node broadcasts a RREQ message to all nodes within the communication range. Each intermediate node which receives the RREQ message appends the source node to the routing table and retransmits it to its neighboring nodes. When the RREQ message reaches the destination node or an intermediate node which has a route to the sink node, the node responds with the RREP message to the source node. Once the source node receives the RREP message, it can transmit data packets to the sink node exploiting the information in the routing table. In the transmission process, the source node transmits the data packet through the main path. However, if the main path becomes unavailable due to failure or other reasons, it transmits the data packet through the alternate path.

IMCMRP [10] was proposed to minimize path interference exploiting four phases: neighbor node identification, cluster head selection, route establishment, and data transmission. In the neighbor identification phase, nearby nodes within the communication range are identified and the transmission cost to neighboring nodes is estimated to establish a path to the sink node. Next, cluster heads are selected based on residual energy and interference degree. When an event occurs, the RREQ message is exploited to request construction of a path to the sink node, and the sink node responds with the RREP message. The source node then completes the multipath construction by sending a confirmation acknowledgment (CACK) message in response to the RREP. The data are divided and sent to the sink node exploiting medium-quality paths for low data rates and high-quality paths for high data rates. After successful data transmission, the sink node notifies the source node, and the source node releases the participating members and the cluster head using the release acknowledgment (RACK) message.

RMR [11] is a radio-disjoint multipath scheme which aims to improve the end-to-end transmission success ratio exploiting multipath. It could increase the lifetime of the network by exploiting the gap called the guard band between the paths reserved for path disjoint. The source node calculates the number of paths based on the required end-to-end transmission success ratio and single-hop transmission success ratio according to the user or applications. Subsequently, it constructs radio-disjoint paths to prevent collision and interference in consideration of the radio range of the sensor node based on the calculated number of paths. The guard band exists between these paths, and it could also be exploited for packet transmission. The odd-numbered packet is transmitted through the constructed paths, and the even-numbered packet is transmitted through the guard band. That is, there is a guard band between the radio-disjoint paths for the transmitting packet, and when transmitting a packet through the guard band, the paths are exploited as a guard band. In this process, intermediate nodes select the next node evenly in the process of transmitting the packet in consideration of the single-hop transmission success ratio, energy consumption and the remaining energy of the neighbor node. Thus, it could prevent specific nodes from being exploited repeatedly. Through this process, RMR could achieve an end-to-end transmission success ratio through multipath transmission while increasing network lifetime.

## 3. Cluster-Disjoint Multipath Routing Protocol

Many applications such as military, medical, and disaster cases require real-time and reliable packet transmission. Multipath routing is exploited to improve reliability; however, it tends to impair real-time transmission due to path construction and path recovery process. Therefore, in this section, we describe a cluster-disjoint multipath routing protocol for real-time and reliable packet transmission. The proposed scheme could be largely categorized as a cluster construction process in network initialization, and a mutlipath transmission process without path construction and path recovery.

### 3.1. Overview

Through this section, we show the overall operation process of the proposed scheme. Each node is aware of its own location by GPS [12] or other techniques [13] and knows the location of the sink node through a network initialization phase [14]. In addition, the nodes keep the information of their neighbor nodes within one-hop range by beaconing.

**Figure 1:**
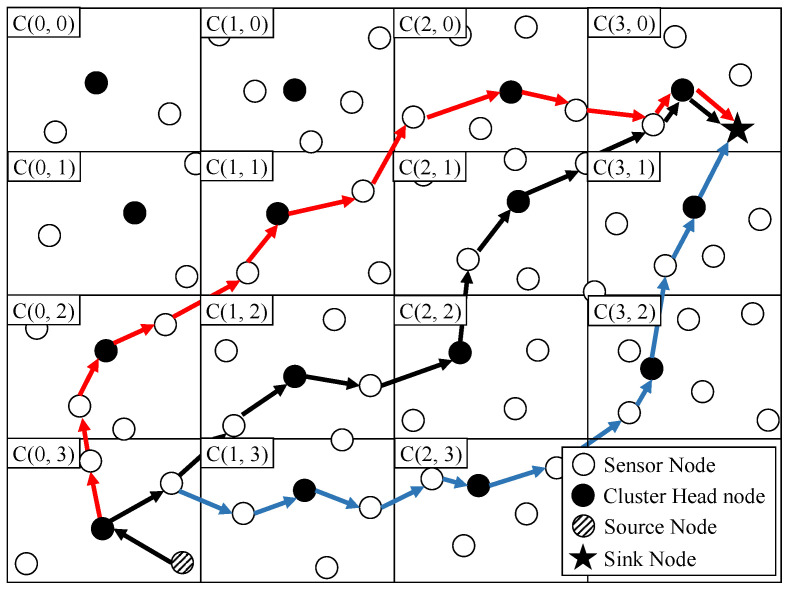
An Overview of Cluster-disjoint Multipath Routing.

In the scenario for the overview, we assume that three paths are required. As shown in Figure 1, the network is divided into a grid cluster of a certain size at the network initialization phase, and each cluster is numbered by an (x, y)-coordinate like C(0, 0), C(0, 1) …, C(x, y). In addition, each cluster has a cluster head elected (see more details in Section 3.2). In cluster C(0, 3), the sensor node detects the event, it delegates the packet to the cluster head. In order to transmit the packet to the sink node located in cluster C(3, 0) through three paths, the cluster head of cluster C(0, 3) selects the cluster C(1, 2) closest to cluster C(3, 0) and relays the packet to the cluster head of the cluster C(1, 2). The cluster head in cluster C(1, 2) selects the next cluster C(2, 2) based on the same criteria as the previous cluster head of cluster C(0, 3), and it repeats this process to transmit the packet to the cluster head where the sink node is located (the black arrow line). For transmissions over different paths, the cluster head of cluster C(0, 3) selects a cluster other than the previously selected cluster C(1, 2) and relays the packet to the cluster head of cluster C(0, 2) (the red arrow line). By performing this process once more (the blue arrow line), the packet could be transmitted to the sink node through three paths. We note that in the above-mentioned packet transmission process with three paths, the construction of multipaths and packet transmission are performed based on a cluster-disjoint approach without the path construction process (see more details in Section 3.3).

### 3.2. Grid Cluster Construction

In this section, we explain the grid cluster construction scheme in the network initialization phase consisting of the following two phases:

- The first phase is detection of the boundary of the network where the sensors are scattered.
- The second phase is division of the detected boundary into appropriate grid clusters.

The sensors in WSNs usually appear in a scattered form [1]. Therefore, although the network does not represent the regular form, it is possible to specify the area of the network through various network boundary detection schemes [15]. However, we focus on grid cluster-based multipath routing, and we assume that the entire network is modeled as a rectangular shape through the network boundary detection process in network initialization.

**Figure 2:**
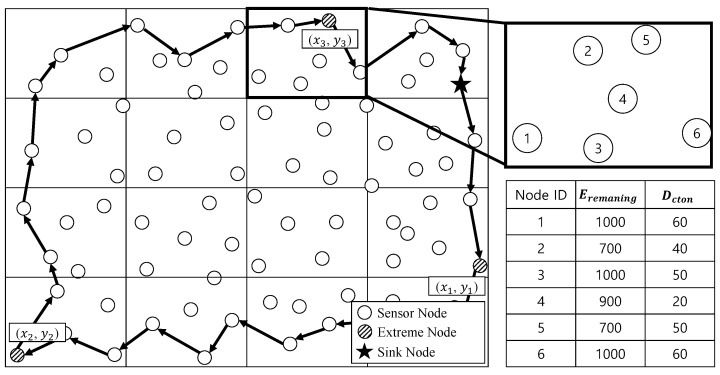
The Extreme Node Detection based on the Boundary Detection Scheme [15].

Figure 2 shows the sink node finding out the network boundaries and calculating a rectangle containing all the sensors. To calculate a rectangle, the following coordinate information is finally stored in the packet relayed during the boundary detection process: (1) the coordinates of the sensor with minimum/maximum x-coordinates and (2) the coordinates of the sensor with minimum/maximum y-coordinates. In Figure 2, sensor ($x_{1}$, $y_{1}$) has maximum x-coordinates, sensor ($x_{2}$, $y_{2}$) has coordinates with minimum x, y-coordinates, and sensor ($x_{3}$, $y_{3}$) has coordinates with maximum y-coordinates.

Algorithm 1 shows the pseudo-code for calculating a rectangle including the whole network. This algorithm is performed in two phases.
**Algorithm 1** Pseudo-code for calculating a rectangle including the whole network 1:Start network boundary detection process exploiting detection packet 2:Packet contains coordinate information to calculate rectangle 3:  4:**if** the packet arrives at node **then** 5:    **if** $x_{min}>x_{node}$ **then** $x_{min}=x_{node}$ 6:    **end if** 7:    **if** $x_{max}<x_{node}$ **then** $x_{max}=x_{node}$ 8:    **end if** 9:    **if** $y_{min}>y_{node}$ **then** $y_{min}=y_{node}$10:   **end if**11:   **if** $y_{max}<y_{node}$ **then** $y_{max}=y_{node}$12:   **end if**13:**end if**          ▹ Detecting the boundary and updating the coordinates14: 15:**if** the packet returned to sink **then**16:   $w=x_{max}-x_{min}$17:   $h=y_{max}-y_{min}$18:**end if**      ▹ The vertex coordinates are $\left(x_{max},y_{max}\right)$, $\left(x_{max},y_{min}\right)$, $\left(x_{min},y_{max}\right)$ and $\left(x_{min},y_{min}\right)$, respectively.

Phase 1 (lines 4–13): The coordinate information is stored in a packet sent by the sink for boundary detection. When a packet reaches each node while traversing the boundary, maximum/minimum coordinate information is compared and updated.

Phase 2 (lines 15–18): When the packet exploited in Phase 1 returns to the sink, the sink could obtain coordinate information of nodes located at the extreme of the network from the packet. Thus, the vertex of the square could be specified exploiting the maximum/minimum x, y coordinates, and the width and height of the square could be calculated.

After the rectangle is calculated, the sink node divides the rectangle into the grid cluster. In this case, the size of the grid cluster depends on the requirements of users and applications. However, it should be wider than the communication range of the sensors to prevent collisions and interference between packet transmission through the grid cluster. The minimum cluster size *L* is calculated through the transmission range of sensor *R* as follows:

$$L=\frac{R}{2\sqrt{2}}$$

After the grid cluster is divided, a cluster head which can represent the grid cluster is elected. The cluster head is responsible for collecting data from its grid cluster and relaying the packet to the sink through the neighboring grid cluster. The criteria for selecting the cluster head are as follows: (1) node location, (2) remaining energy capacity. First, the cluster head is responsible for relaying the packet from the grid cluster, the node should have the largest amount of energy. In addition, since it relays efficiency to the neighboring grid cluster, the location of the node is considered to be the second factor.

The equation for calculating the priority *P* for the cluster head considering remaining energy $E_{remaining}$, total energy amount $E_{total}$, and distance from center of each grid cluster $D_{cton}$ is as follows:

$$P=E_{remaing}/E_{total}*\alpha +\frac{D_{cton}}{R}*\beta$$

In the above equation, α and β are user-defined variables, respectively. They could be used arbitrarily to assign importance to each factor according to the requirements of the user or applications. In Figure 2, the priority of each node is as follows, in the bold grid (in this case, it is assumed that the total energy $E_{total}$ is 1000 and the transmission radius *R* is 50 m). Assuming that the lower the *P* value, the higher the priority, it is in the order of Node 4 (*P* = 1.3), Node 2 (*P* = 1.5), Node 5 (*P* = 1.7), Node 3 (*P* = 2), Node 1 (*P* = 2.2), and Node 6 (*P* = 2.2).

Nodes 1, 3, and 6, which have high remaining energy, might seem the most appropriate candidates; however, if the distance from the center of each grid cluster is far, they could not be appropriate as candidates. For example, if Node 3 is selected as the cluster head, Node 5 would be waste that has to be delivered to Node 3 via Node 4 to transmit the packet to the neighboring grid cluster.

### 3.3. Cluster-Disjoint Multipath Packet Transmission

In this section, we describe a process of transmitting a packet through a multipath based on the grid cluster. The packet transmission process consists of the following three phases.

- In the first phase, the source node which detected the event delegates the packet to the cluster head of its grid cluster.
- The cluster head calculates the required number of paths based on the requirement of application or the user in the second phase.
- Finally, the cluster head branches by the required number of paths and transmits the packet.

The source node which detected the event delegates the packet to the cluster head of the grid cluster to which it is assigned. The cluster head which is entrusted with the packet transmission calculates the required number of paths to satisfy the needs (e.g., end-to-end transmission success ratio) of application or the user. The required number of paths is calculated based on the single hop transmission success ratio and a required end-to-end transmission success ratio [11]. After calculating the required number of paths, the cluster head exploits the grid clusters located on the straight line to the grid cluster where the sink is located as a first path. The other paths are composed of grid clusters surrounding the first path.

**Figure 3:**
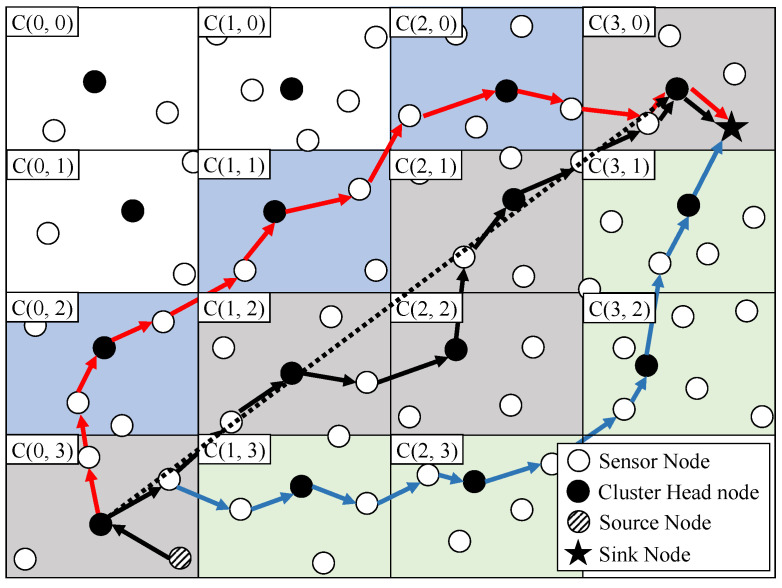
An Example of Cluster-disjoint Multipath Packet Transmission.

Figure 3 shows the data transmission process exploiting the cluster-disjoint multipath. As described above, the source node which detects the event delegates the packet to the cluster head of grid cluster C(0, 3) to which it belongs. The cluster head calculates a straight line to grid cluster C(3, 0) where the sink node is located in order to construct the first path (the black dot line in Figure 3. The cluster head could find out which grid cluster to pass through to transmit the packet to the sink node. In Figure 3, the black dot line penetrates the grid cluster represented in gray, and these grid clusters can become the first path. However, in the case where data can be sent from grid cluster C(0, 3) to grid cluster C(1, 2) located at the diagonal without passing through cluster C (1, 3), although a black dot line penetrates the grid cluster, it does not exploit it as part of first path.

To deal with a case of more paths required according to the requirement of application or the user, the cluster head which participated in the first path in the process of transmitting the packet informs the neighboring grid cluster that it is participating in the first path. For example, the cluster head of grid cluster C(1, 2) which received the packet from the cluster head of grid cluster C(0, 3) informs the cluster heads of grid clusters C(1, 1), C(0, 2), C(2, 2) and C(1, 3) that it is participating in the first path. Through this process, when the cluster head of grid cluster C(0, 3) transmits the packet exploiting other paths based on grid cluster C(0, 2) or C(1, 3), cluster head of grid clusters C(0, 2) or C(1, 3) could prevent duplicate path selection. That is, since cluster head of grid cluster C(0, 2) already knows C(1, 2) has participated in the first path, the cluster head of grid cluster C(0, 2) selects grid cluster C(1, 1) as the next cluster. Similarly, in the case of grid cluster C(1, 1), since grid cluster C(2, 1) participated in the first path, cluster head of grid cluster C(1, 1) selects grid cluster C(2, 0) as the next cluster to transmit the packet. In the same manner as in the above process, the cluster head of grid cluster C(1, 3) transmits the packet through grid clusters C(2, 3), C(3, 2) and C(3, 1). Finally, to deal with the last mile delivery, the grid cluster in which the sink is located does not indicate that it is participating in any path. Thus, the cluster head of the neighboring grid cluster C(2, 0) could forward the packet to the sink in grid cluster C(3, 0). However, if the packet can be directly transmitted without relaying through the cluster head of grid cluster C(3, 0), such as in the case of grid cluster C(3, 1), the packet is directly transmitted to the sink.

The cluster-disjoint multipath constructed by the above process is exploited until the last packet is delivered. In the process of transmitting the packet, each packet can be forwarded through different nodes within the same cluster. Finally, the multipath is released in the process of transmitting the last packet. Now, we describe the process of selecting the next intermediate node to transmit the packet from the previous cluster head to the next cluster head. The process by which a cluster selects the next intermediate node depends on whether there is a sink node in the neighborhood. If there is a sink node, the packet is forwarded directly to the sink node to complete the packet transmission without selecting another intermediate node. (In Figure 3, the cluster head at C(3, 1) relays the packet to the sink node at C(3, 0)). However, when it is necessary to select the next intermediate node to relay data to the sink, a similar criterion to selecting a cluster head is applied. In other words, nodes with higher remaining energy that are closer to the sink are given priority in the selection process. The equation for calculating priority *P* for the cluster head considering the remaining energy $E_{remaining}$, the total energy amount $E_{total}$, and the distance from the intermediate node to sink node *D* is as follows:

$$P=E_{remaing}/E_{total}*\alpha +D*\beta$$

In the above equation, α and β are user-defined variables that could be used if the user wants to assign more weight to energy or distance. The intermediate node selected based on the equation selects the next intermediate node depending on whether there is a cluster head in the neighborhood. If there is a cluster head, the packet directly forwards to the cluster head without selecting another intermediate node to complete packet transmission (in Figure 3, the intermediate node at C(1, 2) forwards the packet to the cluster head at C(2, 2)). However, if it is necessary to select the next intermediate node to forward the packet to the next cluster, the process is repeated and the next intermediate node is selected (in Figure 3, the intermediate node at C(0, 3) selects the next intermediate node to forward the packet to the cluster head at C(1, 2)).

## 4. Performance Evaluation

In this section, we present the simulation results for FD-AOMDV, RMR, and the proposed scheme. FD-AOMDV is a scheme that constructs a node-disjoint multipath through a route discovery phase which exploits RREQ flooding and the RREP response. RMR is a scheme to achieve high reliability based on radio-disjoint multipath routing. In particular, it increases the network lifetime by exploiting different paths for each packet transmission using guardbands located between paths.

In Section 4.1, we explain the simulation environments and evaluation factors. The simulation results according to the end-to-end distance are provided in Section 4.2, and the results according to the single-hop transmission success ratio are provided in Section 4.4.

### 4.1. Simulation Environments

We simulate and analyze the existing protocols and the proposed scheme using the NS-3 simulator. Table 1 provides the detailed simulation environments of our simulation. The nodes are placed in a terrain of a 1000 m × 1000 m area. A total of 100 nodes are placed in the form of a grid, and the remaining 900 nodes are deployed randomly. The performance of each node follows the MICA2 specification. The transmit and receive power consumption values of the node are 24.92 and 19.72 mJ per one byte, respectively. The simulations are performed 30 times, and the graphs represent the average result of the simulations. The evaluation factors and terms are described as follows:

− End-to-end distance is defined as the linear distance between the source and the sink.
− Single-hop transmission success ratio is defined as the average transmission success ratio between neighboring nodes.
− First packet arrival time is defined as the time between the source detecting the event and the arrival of the first packet sent to the sink.
− End-to-end transmission success ratio is defined as the number of packets received by the sink relative to the number of packets sent by the source.
− Energy consumption is the sum of the energy consumption of the nodes that participated in the packet transmission process.

### 4.2. Simulation Results by End-to-End Distance

In this section, we present the performance evaluation results while varying the end-to-end distance under the single-hop transmission success ratio of 90% (from 85% to 95%) to investigate its effect on the first packet arrival time, end-to-end transmission success ratio, and energy consumption.

**Figure 4:**
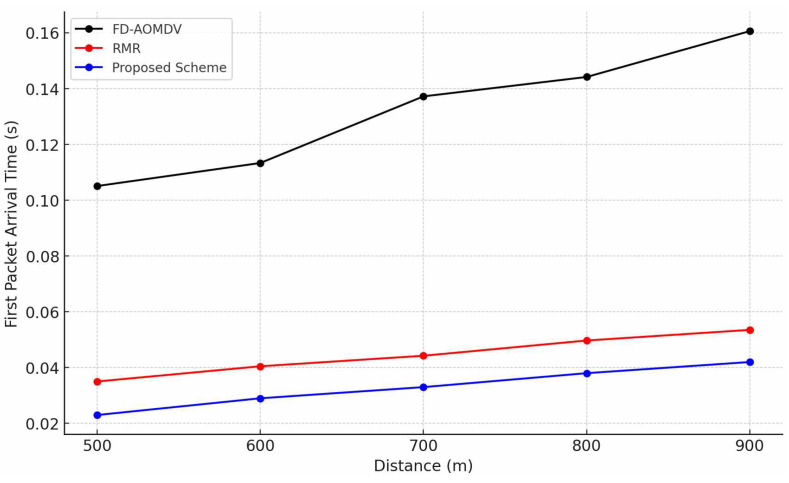
First Packet Arrival Time by End-to-End distance.

Figure 4 shows the first packet arrival time of each protocol according to end-to-end distance. FD-AOMDV has a slower first packet arrival time compared to RMR and the proposed scheme because they both perform path establishment through RREQ message transmission from a source node that detects the event to the sink node and the RREP reply from the sink node. RMR and the proposed scheme are relatively fast due to the immediate computation and route establishment of the source node and the grid cluster head node without the route establishment process based on RREQ and RREP messages. The proposed scheme exploits a pre-calculated grid cluster structure without complex computation at the cluster head and immediately forwards the packet by branching as multipaths as required to satisfy the end-to-end transmission success ratio. RMR does not exploit RREQ or RREP messages; however, it requires additional computational time to construct multipaths. Therefore, the proposed scheme has a slightly faster first packet arrival time compared to RMR.

**Figure 5:**
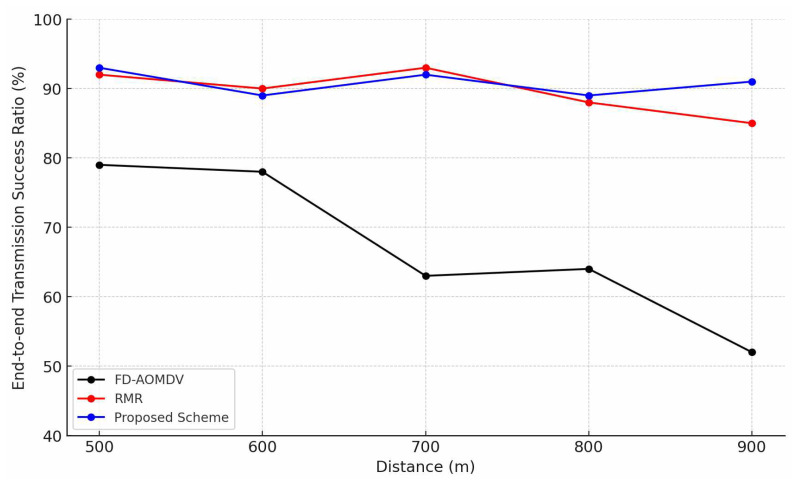
End-to-end Transmission Success Ratio by End-to-End distance.

Figure 5 shows that the end-to-end transmission success ratio of each protocol depends on the end-to-end distance. Each protocol could construct multipaths to achieve the required end-to-end transmission success ratio. However, in case of FD-AOMDV, since it focuses on the node-disjoint of each path, the transmission success ratio is significantly lower than that of other schemes due to collisions and interference between each path in simultaneous transmission exploiting multipaths. For the other two schemes, if the end-to-end distance is short, a sufficient number of paths can be constructed to achieve the required end-to-end transmission success ratio. However, if the required number of paths increases as the distance between the source and sink increases, the RMR cannot have sufficient width for guardband shifting, and the transmission success ratio decreases. In other words, radio-disjoint path construction is achieved, as well as equal utilization of nodes by guardband shifting. However, a larger routing area is required than that in the proposed scheme to achieve the same performance aspect of the end-to-end transmission success ratio. Therefore, as the end-to-end distance increases, RMR shows a lower transmission success ratio than the proposed scheme.

**Figure 6:**
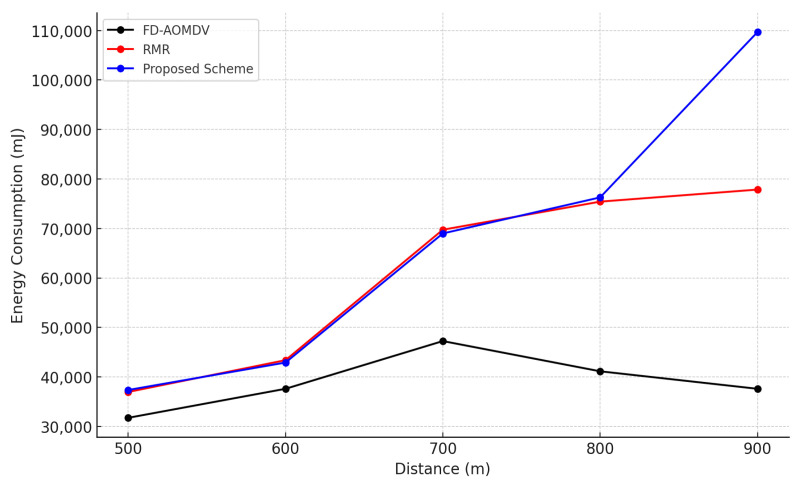
Energy Consumption by End-to-End distance.

Figure 6 shows the energy consumption of each protocol according to end-to-end distance. Energy consumption is a factor that is directly affected by the transmission and reception of packets between nodes, and the proposed scheme and RMR shows an increase in energy consumption as a result of constructing a large number of paths and forwarding packets as the distance changes. However, as mentioned earlier in the end-to-end transmission success ratio, as the distance increases, RMR no longer constructs a sufficient number of paths when the end-to-end distance exceeds a certain distance, and the total number of packet transmissions is reduced compared to that in the proposed scheme. Therefore, the energy consumption does not increase anymore. In the case of FD-AOMDV, although it might construct a sufficient number of paths as required, the energy consumption is relatively low due to transmission failures caused by collisions and interference during simultaneous transmission over the node-disjoint multipath.

### 4.3. Simulation Results by Single-Hop Transmission Success Ratio

In this section, we present the performance evaluation results while decreasing the single-hop transmission success ratio under the end-to-end distance of 1000 m to investigate its effect on the end-to-end transmission success ratio and energy consumption.

**Figure 7:**
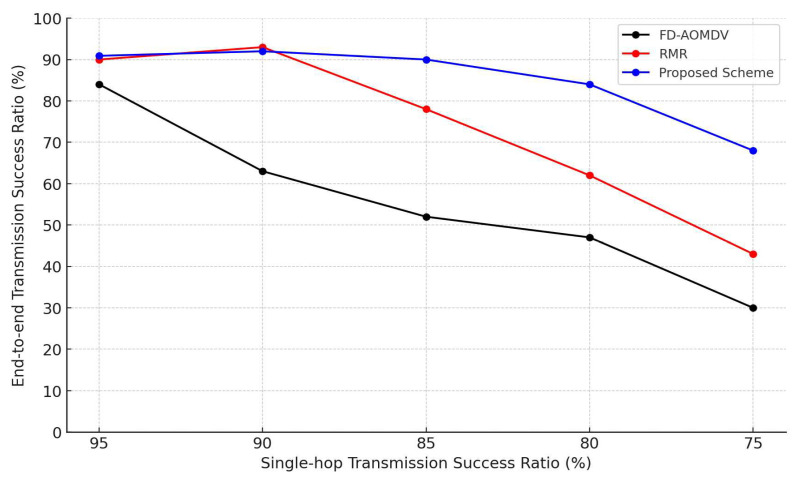
End-to-end Transmission Success Ratio by Single-hop Transmission Success Ratio.

Figure 7 shows end-to-end transmission success ratio of each protocol according to the single-hop transmission success ratio. For all protocols, the end-to-end transmission success ratio decreases as the single-hop transmission success ratio decreases. In particular, FO-AOMDV further reduces the end-to-end transmission success rate due to collisions and interference between nodes located close to each path. The other two schemes, RMR and the proposed scheme, also show a gradual decrease in the end-to-end transmission success ratio as the single-hop transmission success ratio decreases. However, RMR, which exploits guardband shifting, requires a wider width to construct the same number of paths compared to the proposed scheme. Therefore, when the required number of paths increases due to the decrease in the single-hop transmission success ratio, a sufficient number of paths cannot be constructed and the end-to-end transmission success ratio decreases earlier than in the proposed scheme.

**Figure 8:**
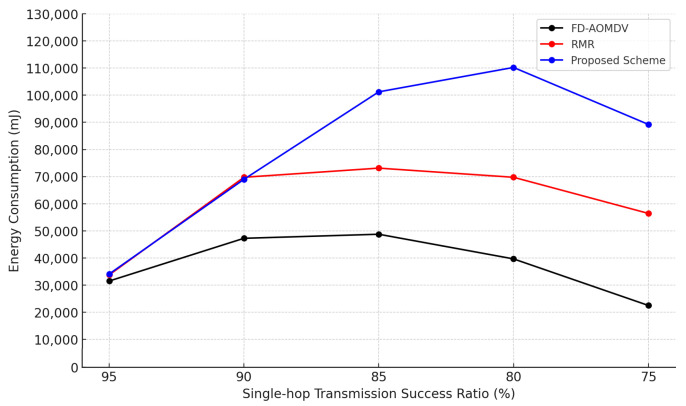
Energy Consumption by Single-hop Transmission Success Ratio.

Figure 8 shows the energy consumption of each protocol according to the single-hop transmission success ratio. In the case of FD-AOMDV, it would construct a multipath to cope with the decreasing single-hop transmission success ratio. However, packet transmission often fails due to interference and collision between paths, resulting in low end-to-end transmission success ratio and low energy consumption. In the case of RMR, similar to the proposed scheme, multipaths are constructed as the single-hop transmission success ratio decreases. However, as mentioned earlier, it could not construct a sufficient number of paths and consumed less energy than the proposed scheme. In the case of the proposed scheme, as the single-hop transmission success ratio decreases, it can construct a larger number of paths than RMR and achieve the required end-to-end transmission success ratio. However, if the single-hop transmission success ratio is extremely degraded, it is difficult to construct a sufficient number of paths and impossible to achieve the required end-to-end transmission success ratio.

Due to this phenomenon, energy consumption is reduced when the single-hop transmission success ratio is low.

### 4.4. Simulation Results under Path Failure

In this section, we present the performance evaluation results when some nodes in the paths are broken while each protocol is transmitting packets. Path failure affects various performance metrics; in particular, it has a significant negative impact on the end-to-end transmission success ratio.

**Figure 9:**
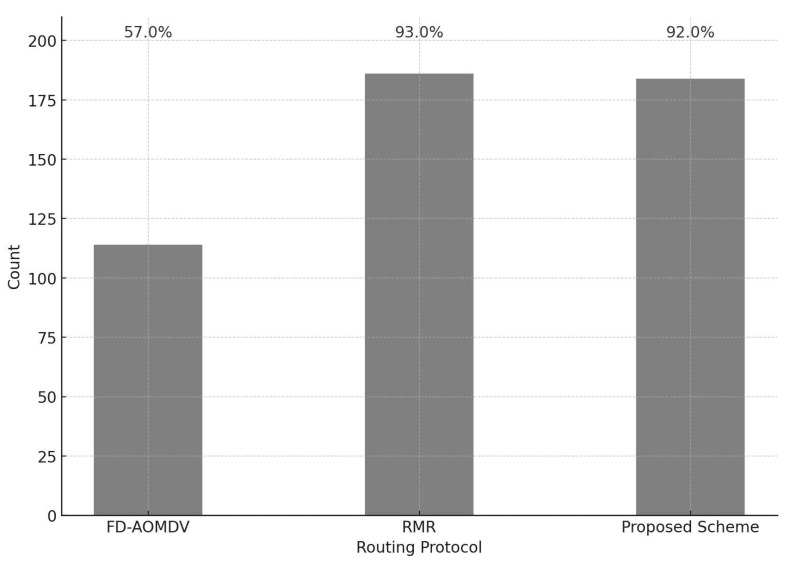
End-to-end Transmission Success Ratio under Path Failure.

Figure 9 shows the end-to-end transmission success ratio when path failure occurs during packet transmission for each protocol. For this purpose, we assume a situation where each protocol constructed a multipath and is ready to transmit a packet. To evaluate the end-to-end transmission success ratio, we calculate the number of packets that arrive at the sink when some nodes in the path are broken during the transmission of 200 packets from the source node. In the case of FD-AOMDV, when path failure occurs, it exploits RREQ and RREP messages to discover the path as in path construction. This results in a lower transmission success ratio until a new path is constructed. In the case of the other two schemes, only the region (or grid cluster) where the packet should be transmitted is specified, and the nodes selected in each transmission process are selected differently according to the criteria of each protocol at each packet transmission. In other words, even if an arbitrary node is damaged, it is immediately replaced by another node in the transmission process and the packet is transmitted. Therefore, both schemes satisfy the required end-to-end transmission success ratio even if path failure occurs.

## 5. Conclusions

In Wireless Sensor Networks (WSNs) exploited to collect information from interest regions, the end-to-end transmission success ratio is one of the most important factors in achieving their main purpose. To improve the end-to-end transmission success ratio, multipath routing protocols, which exploit two or more transmission paths to deliver data, are typically exploited. However, existing schemes suffer from collisions and interference in the transmission process, or time delay and energy wastage due to pre-path configuration and path recovery. Therefore, we propose a cluster-disjoint multipath routing protocol to overcome the above limitations. In the proposed scheme, the network is divided into network clusters of a certain size during the network initialization phase. During packet transmission, each path is determined and transmitted on a cluster-by-cluster basis. Therefore, no path construction time is required for data transmission. In addition, even if some sensors are damaged during the transmission process, data could be delivered immediately via other sensors in the cluster. Therefore, in order to recover the damaged path, time is not require. As a result of performance evaluation, since the proposed scheme does not require a processes such as path construction and recovery based on cluster-based multipath transmission, the proposed scheme is more advantageous in terms of transmission delay and end-to-end transmission success ratio than existing multipath routing protocols.

## Figures and Tables

**Table 1:** Simulation Environment Setting.

Parameter	Value(s)
Routing protocols	FD-AOMDV, RMR, Proposed scheme
Terrain	(1000 m, 1000 m)
End-to-end distance	about 700 m
The number of nodes	1000 nodes Uniform and Random placement
Transmission range (m)	100 m
MAC protocol	802.15.4 MAC
MAC layer	CSMA/CA
Energy consumption (Tx)	24.92 mJ per 1 byte
Energy consumption (Rx)	19.72 mJ per 1 byte
Required end-to-end transmission success ratio	90%
Average Single-hop transmission success ratio	90% (85∼95%)
